# Development of a health‐related quality‐of‐life assessment tool for equines with pituitary pars intermedia dysfunction

**DOI:** 10.1111/evj.14513

**Published:** 2025-05-02

**Authors:** Aline Bouquet, Christine Nicol, Edward J. Knowles, Imogen Schofield, Nicola J. Menzies‐Gow

**Affiliations:** ^1^ Department of Clinical Science and Services The Royal Veterinary College Hertfordshire UK; ^2^ Department of Pathobiology and Population Sciences The Royal Veterinary College Hertfordshire UK; ^3^ Bell Equine Veterinary Clinic Kent UK; ^4^ CVS Group plc Norfolk UK

**Keywords:** geriatric, horse, PPID, quality of life, welfare

## Abstract

**Background:**

Clinical signs of pituitary pars intermedia dysfunction (PPID) are frequently mistaken for ‘normal’ ageing and may not be optimally assessed. Objective quality of life (QoL) assessment could improve clinical decision‐making.

**Objectives:**

To develop an owner‐reported health‐related quality‐of‐life (HRQoL) assessment tool for equines with PPID. To assess factors associated with HRQoL scores.

**Study Design:**

Quantitative, cross‐sectional study.

**Methods:**

HRQoL tool development followed a standard psychometric process of item (any aspect of PPID and its management that could impact QoL) identification (following interviews with veterinarians, owners and clinical record reviews), selection (online owner questionnaire) and refinement (statistical analyses; chi‐squared and Cronbach's alpha). General Linear Models were used to identify factors associated with HRQoL scores.

**Results:**

Forty‐two items associated with PPID were identified. Thirty‐seven items were selected for the online questionnaire. In total, 612 complete responses (*n* = 343 PPID and *n* = 269 non‐PPID horses) were obtained. Through stepwise statistical item refinement, 24 items remained in the final HRQoL tool (overall Cronbach's *α* = 0.835). HRQoL scores ranged from 0 (best) to 1 (worst) QoL. Median (interquartile range) HRQoL scores were 0.33 (0.22–0.44) and 0.20 (0.14–0.27) for PPID and non‐PPID horses respectively. HRQoL scores for all horses were worse if they had PPID (*p* < 0.001) or other chronic medical conditions and were older (*p* < 0.015). For PPID horses specifically, HRQoL scores were also worse if they had other chronic medical conditions (*p* = 0.02), but HRQoL scores were not associated with current PPID treatment (treated vs. untreated horses with a PPID diagnosis), bodyweight, age, breed, sex or years since diagnosis.

**Main Limitations:**

Limited numbers of untreated PPID horses.

**Conclusion:**

The HRQoL tool is valid and reliable for use in horses with PPID and can be applied in further research. PPID horses with another chronic disease had worse HRQoL scores, which should be considered in other studies evaluating disease impact.

## 1 INTRODUCTION

Animal welfare results from the balance between the positive and negative affective (emotional) states that are generated by nutritional, environmental, health and behavioural interactions.^1^, ^2^, ^3^ In turn, the welfare state of an animal integrated over an extended period of time reflects its overall quality of life (QoL).^4^, ^5^ Understanding the impact of specific diseases on welfare and QoL is an important goal. Pituitary pars intermedia dysfunction (PPID) is a common neurodegenerative disease in older horses, associated with clinical signs such as weight loss, muscle wastage and lethargy,^6^, ^7^ and in up to 50% of animals, laminitis.^7^ PPID has the potential to negatively impact welfare and QoL due to chronic pain associated with laminitis,^6^, ^7^ the significant interventions that can be employed to reduce laminitis risk, for example, restricted movement, foot treatment and changes to diet and exercise, and the possibility that some owners do not seek advice or manage the full range of clinical signs associated with PPID^6^, ^7^, ^8^, ^9^, ^10^ due to mistaking them for normal ageing.

The duration of time that an animal experiences poor QoL is an important factor and a key consideration when making decisions regarding medical treatment and/or euthanasia.^4^ Veterinarians typically assess QoL subjectively during day‐to‐day decision‐making. However, although objective QoL assessment is well established in human medicine for monitoring the effects of pain, chronic diseases and age‐related changes,^11^ only a limited number of QoL assessment tools are currently available in the veterinary context (e.g., for cats,^12^ dogs^13^ and zoo animals^14^). Therefore, more objective quantification of QoL, ideally informed by owner input because they are best placed to detect subtle deviations in behaviour and physical health,^13^, ^15^ could be beneficial in optimising decision‐making in practice and providing a validated outcome measurement for future clinical research. Indeed, the British Veterinary Association's Animal Welfare Strategy recently highlighted the use of practice‐based QoL assessments within its priority areas.^16^

In horses, a limited number of welfare assessment tools are available for different contexts, such as working equids,^17^ free‐roaming horses,^18^ and single‐stabled horses^19^ or on‐farm managed horses,^20^ but these are not directly applicable to most equids with PPID. Welfare assessment tools differ, and some do not attempt, or lack the measures, to evaluate the impact of disease on affective state (e.g., the Horse Welfare Assessment Protocol [HWAP]^20^). In contrast, the Standardised Equine Based Welfare Assessment Tool (SEBWAT)^17^ includes animal‐based measures that feed into an evaluation of affective states; however, the focus on working equids is not applicable to the general PPID horse population. Howard et al.^15^ recently published a preliminary validation of an inventory for owner assessment of osteoarthritis, but this had a specific focus on the impact of pain on QoL. Additionally, none of the existing horse welfare assessment protocols assess QoL over an extended period. Researchers have previously suggested the steps needed for QoL‐based decision making,^4^, ^21^, ^22^ but there are no validated tools available that cover all aspects of welfare.

This study aimed to develop a novel health‐related quality‐of‐life (HRQoL) assessment tool for equids with PPID, based on owner assessment of horses in their home environment including their judgements of affective state. A secondary objective was to test for factors that are associated with the HRQoL score obtained from the developed tool.

## 2 MATERIALS AND METHODS

To develop the HRQoL tool, a standard psychometric process of item identification, selection and refinement^13^, ^23^, ^24^ was followed. An item was defined as any aspect of PPID and its management that could impact a horse's QoL. Various individual items could collectively assess a construct (‘domain’) and each item was designed to measure a different facet of the domain. This study was approved by the Social Science Research Ethical Review Board of the Royal Veterinary College, University of London (URN SR2022‐0166).

### 2.1 HRQoL tool development

#### 2.1.1 Item identification

Items were identified using a variety of sources. First, face‐to‐face and telephone semi‐structured interviews were conducted on a convenience sample with 12 veterinarians from a large multidisciplinary equine referral hospital and first‐opinion ambulatory practice (eight first‐opinion veterinarians, two internal medicine specialists and two orthopaedic surgeons), a technical adviser from a pharmaceutical company (Boehringer Ingelheim) and 10 owners of aged horses with PPID (clients of the first‐opinion ambulatory practice). The semi‐structured interviews were conducted using a list of guided, open‐ended questions in general speech regarding the possible effects of PPID on QoL,^13^ with all the answers transcribed for qualitative interpretation. Second, a broader overview of potential items was identified from a review of the relevant literature, a canine Cushing's syndrome QoL tool,^13^ and 20 randomly selected electronic patient records from equines with PPID^13^ from one large UK first‐opinion ambulatory practice and equine referral hospital. In total, 42 items were identified through this process within seven domains (demeanour, appearance, condition, health, appetite, ingestion and management).

#### 2.1.2 Item selection

A draft questionnaire was developed with all 42 identified items. The wording of the questions was based on phrases and words most commonly used by horse owners during the item identification process. The questionnaire was piloted on a convenience sample with veterinarians from a large multidisciplinary equine referral hospital and first‐opinion ambulatory practice, ten client horse owners of the first‐opinion ambulatory practice with and without PPID horses, and additional specialists in animal behaviour and welfare based at a Veterinary Medicine University to identify vague, misleading or missing questions that required revision prior to launching the questionnaire. Five questions were removed due to similarity with other questions. The questions were further reworded for clarification and ease of interpretation based on feedback from the testers. Ultimately, 37 questions were included in the online questionnaire. The final questionnaire (Survey S1) was uploaded to an online survey tool (SurveyMonkey). The questionnaire was targeted internationally at owners of aged horses with and without PPID. The survey was promoted via university and commercial practice websites and social media.

The questionnaire requested background information on the horse's age, breed, sex, bodyweight, height, boarding type, usage, PPID test date, PPID diagnosis (owner‐reported) and treatment, as well as other chronic medical conditions. Owners were first asked to describe their horse's current overall QoL using a Likert scale ranging from ‘very poor’ (5), ‘poor’ (4), ‘neither good nor poor’ (3), ‘good’ (2), to ‘very good’ (1). The following questions asked owners to describe the frequency of each item potentially impacting their horse's QoL over the last month. Responses were assigned the highest score for answers occurring ‘all the time’ or ‘strongly agree’ and lowest scores for ‘never’ or ‘strongly disagree’ for negatively phrased questions, whereas scores were reversed for positively phrased questions. Responses of ‘I have not been able to observe this’ were assigned a middle score to avoid falsely increasing or decreasing the final score (see Table 1).

**TABLE 1:** Final items included in the PPID‐QoL tool after item refinement process.

Item number	Domain	Question	Scoring
1	Demeanour	My horse is dull, depressed, sad and/or withdrawn	All the time (3), often (2), occasionally (1), never (0)
2	Appearance	My horse has a long and thick coat	All over (3), over most of the body (2), in places (1), not at all (0)
3	Appearance	The coat of my horse is patchy	All over (3), over most of the body (2), in places (1), not at all (0)
4	Appearance	The coat of my horse is bristly	All over (3), over most of the body (2), in places (1), not at all (0)
5	Appearance	My horse looks healthy	All the time (0), often (1), occasionally (2), never (3)
6	Appearance	My horse looks older than he/she is	All the time (3), often (2), occasionally (1), never (0)
7	Condition	My horse has lost their topline over the past 6 months	Strongly agree (4), slightly agree (3), neither agree nor disagree (2), slightly disagree (1), strongly disagree (0)
8	Condition	My horse has lost weight over the past 6 months	Strongly agree (4), slightly agree (3), neither agree nor disagree (2), slightly disagree (1), strongly disagree (0)
9	Condition	My horse has a big belly but is thin	Strongly agree (4), slightly agree (3), neither agree nor disagree (2), slightly disagree (1), strongly disagree (0)
10	Health	My horse has laminitis flare‐ups	All the time (3), often (2), occasionally (1), never (0)
11	Health	My horse has skin problems	All the time (3), often (2), occasionally (1), never (0)
12	Health	My horse has eye problems	All the time (3), often (2), occasionally (1), never (0)
13	Health	My horse moves around freely and without any pain	All the time (0), often (1), occasionally (2), never (3)
14	Appetite	My horse is fussy with and/or off their hard feed	All the time (3), often (2), occasionally (1), never (0), I have not been able to observe this (1.5)
15	Appetite	My horse has no appetite (hay and other forage)	All the time (3), often (2), occasionally (1), never (0), I have not been able to observe this (1.5)
16	Appetite	My horse drinks a lot	All the time (3), often (2), occasionally (1), never (0), I have not been able to observe this (1.5)
17	Ingestion	I struggle to get my horse to eat the medication(s) and/or supplement(s) in their bucket feed, and I have to feed it in a treat or similar	All the time (3), often (2), occasionally (1), never (0), my horse does not receive any meds/suppl.
18	Management	My horse lives a more restricted life than most other horses	Strongly agree (4), slightly agree (3), neither agree nor disagree (2), slightly disagree (1), strongly disagree (0)
29	Management	My horse is still able to do all the activities that they used to enjoy and do	Strongly agree (0), slightly agree (1), neither agree nor disagree (2), slightly disagree (3), strongly disagree (4)
20	Management	Because of the special needs of my horse and the additional care I give to my horse, my social and/or working life is affected	Strongly agree (4), slightly agree (3), neither agree nor disagree (2), slightly disagree (1), strongly disagree (0)
21	Management	I struggle to manage the health of my horse	Strongly agree (4), slightly agree (3), neither agree nor disagree (2), slightly disagree (1), strongly disagree (0)
22	Management	I worry about the future of my horse	Strongly agree (4), slightly agree (3), neither agree nor disagree (2), slightly disagree (1), strongly disagree (0)
23	Management	I worry about the health and wellbeing of my horse and have considered euthanasia	Strongly agree (4), slightly agree (3), neither agree nor disagree (2), slightly disagree (1), strongly disagree (0)
24	Management	I worry about the costs of the ongoing medical treatments that my horse needs to stay healthy	Strongly agree (4), slightly agree (3), neither agree nor disagree (2), slightly disagree (1), strongly disagree (0)

#### 2.1.3 Post‐survey item refinement

To develop the finalised HRQoL tool, the questions were refined based on a statistical analysis of the responses, as previously described.^13^ Incomplete item question responses were excluded from the statistical analyses. Raw data were collated and standardised in Microsoft Excel. All standardised variables of interest and additional background data were imported into IBM SPSS (version 28) for statistical analyses. Statistical significance was set at *p* < 0.05. Normality was assessed based on the Shapiro–Wilk test; results are presented as mean ± (SD) for normally distributed variables or median (IQR) for non‐normally distributed variables. Chi‐squared analysis was performed for each item to compare the responses of horses with and without PPID. Items that were significantly (*p* < 0.05) different between the two groups were retained, as these were considered specific to the impact of PPID on HRQoL. Cronbach's alpha was used for responses from owners with PPID horses to assess the internal consistency (i.e., reliability of questions to measure the same latent concept, here HRQoL) of retained questions. An inter‐item correlation matrix summarised the extent to which individual item responses correlated with all included items. Questions with high correlations (r > 0.60) within the same domain (key area that encompasses QoL) were deemed to record the same information and, therefore, falsely raise the internal consistency of the tool. Questions with low corrected item‐total correlations (r < 0.30) were removed if their removal increased the overall Cronbach's alpha coefficient. Internal consistency for retained questions was considered appropriate if *α* > 0.70.

### 2.2 Interpretation, validation and reliability of the HRQoL tool

**FIGURE 1:**
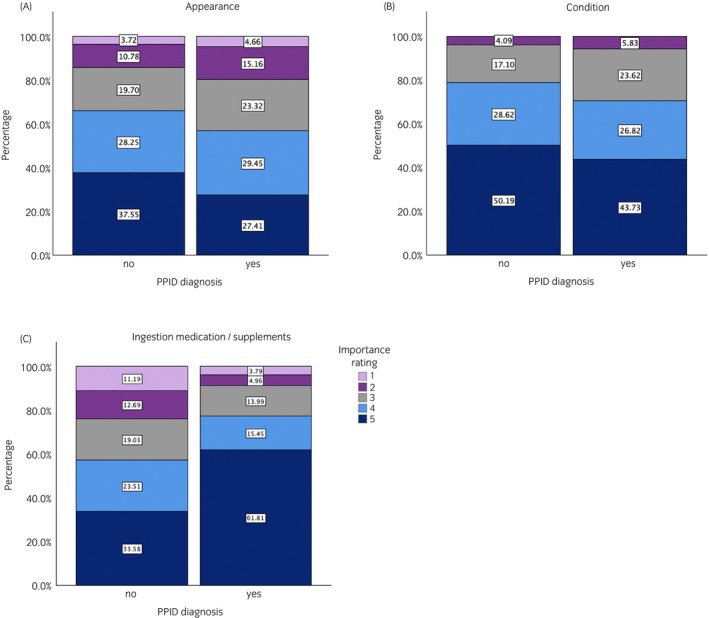
Percentage distributions of domain importance rating in determining overall QoL between PPID horse owners and non‐PPID horse owners for domains: appearance (A), condition (B) and ingestion medication/supplements (C). 1 = not important; 2 = slightly important; 3 = important; 4 = fairly important; 5 = very important.

At the end of the questionnaire, owners were asked to rate the importance of each of the seven item domains (demeanour, appearance, condition, health, appetite, ingestion and management). This information was used to weight each item with the median corresponding domain importance score for the final HRQoL score calculations (Figure 1). The importance rating was scored on a five‐point scale ranging from ‘not important at all’ (1), ‘slightly important’ (2), ‘important’ (3), ‘fairly important’ (4) and ‘very important’ (5). The order in which each domain importance question was asked was randomised for each respondent. A Kendall's tau c test measured the degree of ordinal association, that is, associations/differences in the distributions of owner‐perceived domain importance ratings in determining their horse's overall owner‐assessed QoL between PPID and non‐PPID horse owners (Table 2). The medians for each domain importance from PPID horse owners were used to weight individual questions, that is, the score of each question was multiplied by the relevant domain weight. The final tool with 24 items was used to produce a HRQoL score. The QoL score ranged from 0 (best possible QoL) to 1 (worst possible QoL) and was calculated as follows:

HRQoL score=∑of the weighted question scores/total maximum score.

**TABLE 2:** Median (IQR) owner‐perceived importance rating of each domain determining a horse's overall QoL based on PPID horse owners and non‐PPID horse owners' responses.

Domain	PPID horse owners	Non‐PPID horse owners
Demeanour	5 (5, 5)	5 (5, 5)
Appearance	4 (3, 5)	4 (3, 5)
Condition	4 (3, 5)	5 (4, 5)
Health	5 (5, 5)	5 (5, 5)
Appetite	5 (4, 5)	5 (4, 5)
Ingestion medications/supplements	5 (4, 5)	4 (3, 5)
Management	5 (4, 5)	5 (4, 5)

*Note*: 3 = important, 4 = fairly important, 5 = very important.

To maximise the accuracy, reliability and reproducibility of the tool and avoid unconscious bias,^25^ the degree of agreement between two owners or an owner and a sharer/carer for the same horse at the same time (inter‐rater reliability, *n* = 8) and the degree of consistency of measurements within one observer (intra‐rater reliability, *n* = 7, two‐week interval) was tested on a subsample of 33 participants who fulfilled the requirements of having a sharer and gave consent to be contacted by the research team. It was specified that for the inter‐rater reliability, participants must complete the questionnaire independently from each other without any communication about it until after completion to avoid bias. Inter‐ and intra‐rater reliability was assessed with the intraclass correlation coefficient (ICC), with values of <0.50 indicating poor reliability, 0.50–0.75 moderate, 0.75–0.90 good and >0.90 excellent reliability.^13^, ^26^ Finally, a Spearman's rank correlation test was used to compare owner QoL assessments (given at the start of the survey) with the calculated HRQoL score and demonstrate face validity of the tool.

### 2.3 Factors predicting HRQoL score

The background data for each horse were used to access factors associated with the final overall HRQoL score. Total sample size for these analyses was *n* = 607 (PPID: *n* = 340; non‐PPID: *n* = 267) due to one missing breed entry and two missing age entries for PPID horses, and one missing breed and one age entry for non‐PPID horses. The variable HRQoL score was not normally distributed based on the Shapiro–Wilk test outputs; thus, the variable was transformed by applying a square root transformation (SQRT). For all models, unless otherwise stated, HRQoL score (SQRT) was entered as the dependent variable. To reduce the multiplicity of *P*‐values and, therefore, Type I error,^27^ only the main effects and two‐way interactions between a fixed factor and a covariate or random factor and a covariate were entered in all models. Scatterplots of unstandardised residuals and standardised predicted values were assessed for homoscedasticity, and the Shapiro–Wilk test was used to assess normality in order to assess the assumptions of statistical tests used. To test for factors affecting HRQoL scores in horses with or without PPID, a univariate General Linear Model (GLM) was used. To test for the effects of PPID or other chronic medical conditions, PPID diagnosis (binary) and other chronic medical conditions (binary) were entered as fixed factors. To test for the effects of breed, sex and body condition, the variables breed, sex and body condition were entered as random factors. Age was entered as a covariate to test the effects of age. To test for factors that affect HRQoL scores in horses with PPID, the dataset was split by PPID diagnosis, and a univariate GLM was run. PPID treatment (binary) and chronic medical conditions (binary) were entered as fixed factors; breed, sex (binary) and body condition were entered as random factors; and age and years diagnosed were entered as covariates.

## 3 RESULTS

### 3.1 HRQoL tool development

#### 3.1.1 Survey completion rate

A total of 687 survey responses were collected, with a completion rate of 88% (612 complete responses). Of these complete responses, 343 were from owners with PPID horses (56%) and 269 were from owners of horses without PPID (44%).

#### 3.1.2 Post‐survey item refinement

Items were removed from the tool if (i) the item was not considered distinctive to the impact of PPID on HRQoL (i.e., Chi‐squared analysis revealed no difference between the responses of PPID and non‐PPID horse owners); (ii) items (responses from PPID horse owners only) were redundant (i.e., highly correlated) or did not measure the same underlying construct (HRQoL) (i.e., poorly correlated).

Using Chi‐Squared test of independence analysis, there was no significant difference in the responses of owners with and without PPID horses for the questions: ‘my horse is moody’ (χ^2^(3) = 0.978, *p* = 0.8), ‘my horse enjoys engaging with other horses’ (χ^2^(3) =2.235, *p* = 0.5), ‘my horse enjoys engaging with myself and other care takers’ (χ^2^(3) = 3.465, *p* = 0.3), ‘when my horse stands in the field, stable or tied up, most of the time their facial expression looks like’ (Grimace scale^28^) (χ^2^(2) = 2.775, *p* = 0.3), ‘my horse is footy’ (χ^2^(3) = 2.669, *p* = 0.5), ‘my horse has bad teeth relative to their age’ (χ^2^(1) = 1.301, *p* = 0.3), ‘I feel there is a strong bond between me and my horse’ (χ^2^(4) = 5.016, *p* = 0.3). These items were removed from the tool because they did not have a distinctive impact on HRQoL in our population.

Using Cronbach's alpha analyses, the responses to ‘my horse is dull, depressed and/or sad’ and ‘my horse has a lack of interest in life and/or is withdrawn’ were highly correlated (*r* = 0.627). The two questions were therefore combined into one ‘my horse is dull, depressed, sad and/or withdrawn’ for the final tool.

The reliability of the tool was further improved by removing five additional questions that were poorly correlated with total items (‘is your horse on non‐prescribed supplements’, *r* = −0.001; ‘is your horse on prescribed medication’, *r* = 0.066; ‘my horse is spooky and unpredictable’, *r* = 0.062; ‘my horse has a cresty neck and other fat pocket deposits’, *r* = 0.150; ‘my horse needs clipping more often’, *r* = 0.199). Each removal improved overall Cronbach's alpha. The question ‘My horse does not eat the medication and/or supplement in their bucket feed, I have to feed it in a treat or similar’ was considered to yield the same information as the question ‘I struggle to get my horse to eat the medication(s) and/or supplement(s)’ by the research team, and these were combined into ‘I struggle to get my horse to eat the medication(s) and/or supplement(s) in their bucket feed, and I have to feed it in a treat or similar manner’ for the final tool. Through this stepwise process of item refinement, the initial count of the 37 items was reduced to 24 for the final PPID‐QoL tool, with an overall Cronbach's alpha of 0.835 (Table 1). The final HRQoL tool took participants about 6 min to complete (without any additional background information questions) as assessed on SurveyMonkey during inter‐ and intra‐reliability testing.

#### 3.1.3 Inter‐ and intra‐observer reliability

Intra‐rater reliability indicated good reliability (*n* = 8, ICC = 0.835, 95% CI 0.174–0.967) using a consistency agreement definition. Inter‐rater reliability indicated excellent reliability (*n* = 7, ICC = 0.915, 95% CI 0.570–0.985) using an absolute agreement definition.

#### 3.1.4 Owner‐rated overall QoL assessment (PPID and non‐PPID horses combined)

**FIGURE 2:**
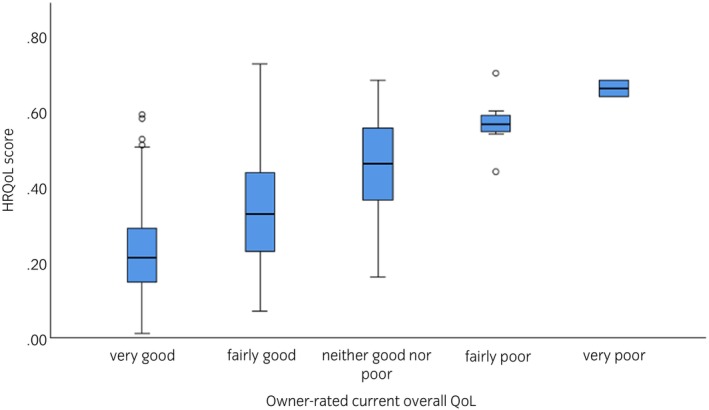
HRQoL scores in horses with and without PPID, separated by current owner‐perceived QoL assessment. Bars represent 95% confidence intervals.

The majority of owners assessed their horses' current QoL as very good (52.0%), followed by fairly good (42.0%). Fewer owners rated QoL as neither good nor poor (4.6%), fairly poor (1.1%) or very poor (0.3%). There was a moderate positive correlation between the owner‐rated overall QoL assessment and the final HRQoL score (*r*_s_(610) = 0.466, *p* < 0.001; Figure 2).

### 3.2 Factors associated with HRQoL score

Differences between the population profile of PPID and non‐PPID horses are shown in Table 3. The most reported other chronic medical conditions in this population were equine metabolic syndrome, arthritis, severe equine asthma, skin conditions such as sweet itch and muscle myopathies.

**TABLE 3:** Population profile differences between PPID and non‐PPID horses.

	PPID horses	Non‐PPID horses
HRQoL score	Median 0.33 (IQR 0.22, 0.44)	Median 0.20 (IRQ 0.14, 0.27)
Age (years)	Mean 24.34 (SD 6.60)	Mean 19.84 (SD 5.16)
Height (hh)	Median 15 (IQR 13.2, 15.3)	Median 15.1 (IQR 14.2, 16.0)
Breed		
Mostly or pure native	48.1%	44.2%
Mostly or pure WB	9.9%	16.7%
Mostly or pure TB	9.3%	16.4%
Mostly or pure Arabian	6.4%	4.1%
Mostly or pure draught	4.4%	5.9%
Cross breed	8.5%	6.7%
Other breed	13.1%	5.9%
Missing	0.3%	
Sex		
Mare	43.7%	35.3%
Gelding	56.0%	63.9%
Stallion	0.3%	0.7%
Body condition		
Very underweight	3.8%	0.7%
Slightly underweight	20.7%	7.1%
Ideal weight	53.4%	41.3%
Slightly overweight	21.0%	46.8%
Very overweight	1.2%	3.7%
Other chronic medical conditions (binary)		
Yes	35.3%	48.0%
No	64.7%	52.0%
Number other chronic medical conditions		
0	39.4%	48.0%
1	46.1%	41.6%
2	11.7%	8.9%
3	2.3%	1.1%
4	0.6%	0.4%
Horse boarding		
DIY (self‐care boarding)	48.4%	51.3%
Partial boarding	4.7%	6.3%
Full boarding	9.9%	9.3%
Other (e.g., at home)	37.1%	33.1%
Previous use		
Competition	44.0%	52.0%
Pet	53.1%	46.8%
Work horse	2.3%	1.1%
Missing	0.6%	
Current use		
Competition	4.4%	15.2%
Pet	95.0%	84.8%
Work horse	0.6%	
Time owned (years)	Median 15 (IQR 8.5, 20)	Median 10 (IQR 5,17)
Years diagnosed (PPID)	Median 2 (IQR 1, 6)	
PPID treatment		
Prascend (Boeringer Ingelheim Ltd., Bracknell UK)	77.0%	
Injectable cabergoline (Bova UK Ltd., London or Bova Aus, Caringbah, Australia; BetPharm, Lexington, USA)	0.9%	
Pergolide compounded	1.5%	
Pergolide paste (Bova UK Ltd., London or Bova Aus, Caringbah, Australia)	1.5%	
Pergoquin (Richter Pharma AG, Wels, Austria)	5.2%	
No veterinary‐prescribed treatment	13.1%	
Other	0.9%	
PPID horse not eating oral medication in bucket feed, requires special treat for medication intake	*N* = 320	
All the time	39.1%	
Often	8.4%	
Occasionally	17.2%	
Never	35.3%	

**FIGURE 3:**
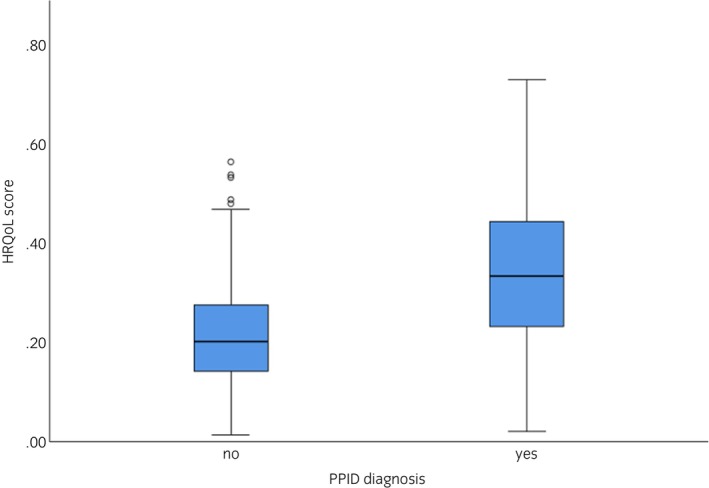
Differences in HRQoL scores between horses without PPID and horses with PPID using non‐transformed data. Bars represent 95% confidence intervals.

**FIGURE 4:**
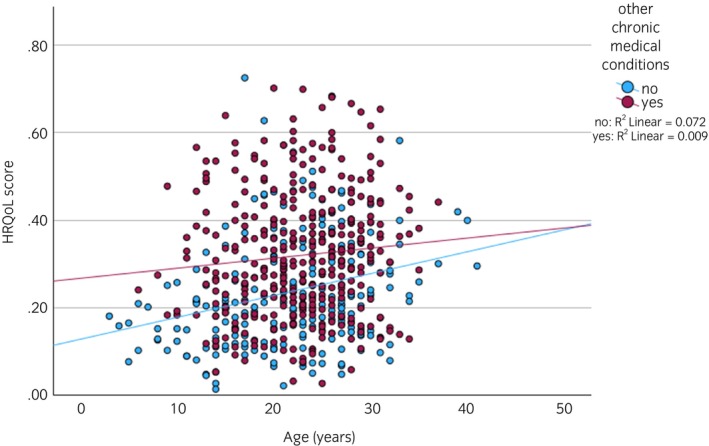
Relationship between horse age and HRQoL score by the presence of other chronic medical conditions. Graph represents non‐transformed data and therefore does not fully reflect GLM run.

When analysing data from all horses, HRQoL scores were significantly worse if they were diagnosed with PPID (*F*_1,579_ = 11.535, *p* < 0.001; Figure 3) or had other chronic medical conditions (*F*_1,579_ = 16.429, *p* < 0.001). However, horses had a significantly worse HRQoL score if they had other chronic medical conditions and were older (interaction term: *F*_1,579_ = 5.926, *p* = 0.015; Figure 4). HRQoL score was not associated with breed, sex or body condition or age as a main effect (Table S1). There were no significant interactions between horse age and PPID diagnosis, breed, sex or body condition (Table S1).

When the PPID horses were analysed as a specific group, HRQoL scores were worse if they also had other chronic medical conditions (*F*_1,285_ = 5.182, *p* = 0.024), but HRQoL scores were not significantly associated with current PPID treatment (i.e., treated vs. untreated horses with a PPID diagnosis), body condition, age, breed, sex or years since diagnosis, or any two‐way interactions between a covariate and a fixed or random factor (Table S2).

## 4 DISCUSSION

This HRQoL tool was developed to quantify the perceived impact of PPID on QoL and identify factors that are associated with HRQoL scores. It is important that QoL assessment tools are reliable, valid and interpretable.^13^, ^29^ Belshaw et al.^29^ point out that many QoL assessment tools for dogs fail to assess these components. The following discussion aims to demonstrate the reliability, validity and interpretability of the developed HRQoL tool.

The final HRQoL tool consisted of 24 items, with good internal reliability and an overall Cronbach's alpha of 0.835. The overall Cronbach's alpha is similar to those previously reported in QoL tools for dogs with Cushing's disease,^13^ osteoarthritis,^30^ and allergic dermatitis,^31^ and slightly lower than the Equine Brief Pain Inventory.^15^ Thus, all items in the current HRQoL tool contribute appropriately and measure the same latent concept: namely, the QoL of horses with PPID. The tool length, in terms of the number of items (24), and the total time taken to complete the questionnaire (ca. 6 min without any additional background information questions) are comparable to other QoL tools.^13^, ^30^, ^31^, ^32^ Therefore, the implementation of this tool could first be applied in future research and further developed into a PPID‐specific QoL assessment tool for use in clinical practice.

The HRQoL tool had good reliability for intra‐rater reliability and excellent reliability for inter‐rater reliability, indicating that in general, two different observers evaluating the same horse at a given time point had better agreement than the same observer evaluating a horse over a two‐week interval. This has also been reported by Schofield et al. for a canine Cushing's disease HRQoL tool.^13^ Within the two‐week time period the QoL of the tested horses may have changed due to disease progression or altered environmental factors. Alternatively, as previously reported,^13^ familiarity with the questions could alter owners' behaviour and attitude towards them. Unfortunately, no comparison can be made to the preliminary validated Equine Brief Pain Inventory for horses with osteoarthritis^15^ because the inter‐ and intra‐rater reliability were not formally assessed; only medians and ranges in individual item scores of owner assessment consistency at a two‐day interval were reported. In a review paper on QoL assessments in dogs, Belshaw et al.^29^ recommended an appropriate time frame of 1–2 weeks for intra‐rater reliability assessment. This recommendation informed the assessment of internal consistency, intra‐ and inter‐rater reliability in the current HRQoL tool.

QoL domains were weighted because not all domains equally impact a horse's QoL or are not perceived by an owner to equally impact their horses' QoL. To establish the appropriate domain weighting to calculate the final HRQoL score, owners were asked how much each domain contributed to determining a horse's QoL. Most horse owners reported that each domain is ‘fairly’ or ‘very important’, although PPID horse owners rated condition and appearance as less important than non‐PPID horse owners, and ingestion of medication as more important. These differences may be due to the clinical signs associated with PPID impacting appearance, such as hypertrichosis and epaxial muscle atrophy, the side effects of veterinary treatment that result in inappetence,^6^ or owners struggling to administer oral medications. Some aspects of ‘Appearance’ and ‘Condition’ may be down‐weighted by PPID horse owners because they can be managed, or because they are no longer highly relevant, for example, hypertrichosis can be managed through clipping, and muscle atrophy may not be an issue for retired horses where saddle fit is no longer an issue. In contrast, PPID owners up‐weighted the ingestion of medication as more important than non‐PPID owners. This may reflect the considerable difficulties that PPID owners reported in orally administering pergolide to their horses. Almost 40% of owners reported that they struggled to feed pergolide all the time and had to hide it in a treat. Complete refusal of oral medication intake can be frustrating for horse owners and may lead to horses not receiving medical treatment if alternatives (e.g., injectable cabergoline) are not appropriate. This could be one reason why a previous study found that 52% of horse owners were non‐compliant with pergolide dosing.^33^ Therefore, depending on the context, PPID horse owners may feel different domains are more or less important but further work is required to validate such perceptions.

Other studies have applied weighting to QoL tools through different approaches, such as item‐weighted impact score.^13^, ^24^, ^34^ For example, Schofield^35^ explored the option for owners to weight the questions on each completion of the developed tool, considering that individualised interpretation of importance may vary. However, the later paper^13^ decided not to weight the tool as overall score reliability was reported to be negatively affected because these individual importance scores proved to be subjective in two ways: (1) disagreements between two owners' views and (2) variations in repeated owner responses within a two‐week period. This impact on reliability was also reported by Browne et al.^36^ As a result, in the development of the present HRQoL tool, each item was weighted using the median domain importance rating of the PPID horse owners, taking an owner consensus view of importance into account. While this approach may not be practical for everyday use for horse owners or veterinarians in practice, here, the aim of weighting was to be as accurate as possible and to best interpret QoL impact. Indeed, Long et al.^4^ highlighted that if the goal of QoL assessment is to inform a decision of euthanasia due to poor QoL, it is not enough to assess a range of items without weighting or prioritising items for an overall QoL score.

The majority of horse owners assessed their horse's QoL as very good (52%) or fairly good (42%), which is in agreement with a previous study that investigated owners‐perception of QoL in geriatric horses,^8^ where 95% of horse owners assessed their horses' QoL to be good or excellent. However, there was only a moderate positive correlation (*r*_s_ = 0.466) between the owner‐assessed overall QoL and HRQoL score, and for many horses with a higher (worse) HRQoL score, their owners indicated that they assessed their horse's current overall QoL to be very good. This is in agreement with the findings of the CushQoL‐pet score and owner‐perceived QoL in dogs with Cushing's disease as previously reported by Schofield et al.^13^ Consequently, the detailed assessment of different domains that reflect the multiple facets of QoL appears to hold more value above a singular direct question for overall QoL assessment.^13^ This discrepancy may have a number of underlying causes. Different owner attitudes towards osteoarthritis in horses have recently been reported.^15^ One viewpoint was that all older horses develop osteoarthritis to some degree, therefore it is normal and does not require veterinary diagnosis, which may lead to untreated chronic pain and as a result reduced QoL. Other owners felt that (because) they took good care of their horses, none of them had arthritis, reflecting a misconception that osteoarthritis is caused by improper management, and it badly reflects on the owner.^15^ A related attitude may be that ‘I provide my horse with a good life; therefore, they must have good QoL’. These varying attitudes may be similarly relevant to PPID. However, this requires further investigation.

In human studies, caregivers do not always accurately assess another human's QoL.^37^, ^38^, ^39^ Therefore, it is expected that the human judgement of a horse's QoL will not be identical to the horse's own appraisal of their life. Despite the well‐meaning care and attention horses receive from their owners, riders and grooms, it is not uncommon that obesity, pain and delayed euthanasia are not recognised and addressed in a timely manner.^40^, ^41^, ^42^, ^43^ This can lead to an unintentional welfare compromise and poor QoL over time. For that reason, if disease‐specific QoL tools can encompass multiple facets that are associated with a particular disease, the QoL may be more accurately assessed. This further reinforces the use of such objective QoL assessment tools. The HRQoL tool can inform owners and veterinarians about an individual horse's QoL, but it does not in itself determine QoL. It allows the tracking of QoL changes over time and ensures that no relevant aspects are overlooked.^4^ Ultimately, the change in score over time, owners' willingness and available resources for interventions should form the basis for any decision‐making with regards to treatment options or ending the individual's life.

In the current study, PPID diagnosis was significantly associated with higher (worse) HRQoL scores. This indicates that QoL quantified by the HRQoL tool is relevant to equine PPID and further validates the tool. However, HRQoL scores were also significantly associated with the presence of other chronic medical conditions, such as equine metabolic syndrome, arthritis, severe equine asthma and skin conditions such as sweet itch, as well as increasing age. The influence of chronic disease on QoL is unsurprising, and it, and its associated signs of pain, are often not recognised by owners,^42^, ^43^, ^44^ leading to poorer QoL. In the current study, the HRQoL score was not associated with horse breed, sex or body condition or age as a main effect. Comparably, the CushQoL‐pet tool for dogs with Cushing's syndrome was also not influenced by dog age or other morbidities.^13^ For PPID horses specifically, HRQoL scores were also worse if they also had other chronic medical conditions. Given that other chronic medical conditions also influence the HRQoL score within a PPID population only, it seems predictable that multiple chronic conditions have a detrimental impact on QoL and thus need to be taken into account when assessing individual horses.

In the current study, there was no effect of PPID treatment on HRQoL scores in individuals with PPID. However, due to the small sample size of untreated PPID horses, caution should be exercised when interpreting these results. The majority of respondents with PPID‐diagnosed horses reported that their horses were treated with pergolide, consistent with the relatively high uptake of medical treatment for PPID horses and owner satisfaction with PPID treatment previously reported.^45^ This may be a contributing factor as to why the variable years since diagnosis did not affect the HRQoL score, despite being a progressive disease,^46^ since clinical signs are under control with current veterinary‐prescribed treatment. Many horse owners mentioned negative effects of the medication on overall demeanour and appetite during item identification interviews. It is therefore possible that any beneficial effects of treatment on QoL were countered by negative side effects of the medication leading to no overall effect. To truly conclude if PPID treatment influences QoL and whether our tool is sufficiently sensitive to detect this, we require (a) a larger sample size of PPID horses not on treatment and (b) ideally to track QoL from the time of diagnosis and start of veterinary‐prescribed treatment over time.

The limitations of this study should be acknowledged. This study may have been influenced by a potential recruitment bias.^47^, ^48^, ^49^ Calls for survey study participants were made through various avenues, such as UK‐based veterinary practices, social media and horse‐riding magazines. This may have targeted people who were interested in sharing, consuming and exchanging information about PPID, particularly on social media, which yielded the majority of survey responses. The survey was only accessible online so participants required internet access to complete it and these participants may not be representative of the general horse‐owner population. Another limitation is that owners had to indicate whether their horse was diagnosed with PPID by a veterinarian or not, but this study did not collect or access veterinary records to confirm the diagnosis and this may have been a source of error. In humans, a healthy volunteer bias has been reported;^50^ therefore, horse owners may have been more invested in completing the survey if they thought their horse has good QoL and may partially explain why most horse owners assessed their horse to have very good or fairly good QoL. Furthermore, test–retest reliability, which assesses the consistency in scoring over a long period of time,^29^ is yet to be completed for this PPID‐QoL tool.

In conclusion, the tool encourages owners and clinicians to consider a range of factors that are likely to impact on the QoL of horses with PPID in a systematic manner. The HRQoL tool shows great promise for use in a clinical setting but needs test–retest reliability assessment and further testing on a larger non‐treated PPID population before it can be deployed in practice. However, the implementation of the HRQoL tool has value, is quick for owners to complete, easy for veterinarians to interpret, and provides an objective method for owners and veterinarians to monitor the QoL over time and to communicate decision‐making in relation to treatment and euthanasia.

## FUNDING INFORMATION

This study was funded by a grant received from CVS Ltd.

## CONFLICT OF INTEREST STATEMENT

E. J. Knowles and I. Schofield are both employed by CVS Ltd.

## AUTHOR CONTRIBUTIONS

**A. Bouquet:** Conceptualization; investigation; writing – original draft; methodology; validation; visualization; writing – review and editing; software; formal analysis; project administration; data curation. **C. Nicol:** Funding acquisition; writing – review and editing. **E. J. Knowles:** Funding acquisition; writing – review and editing. **I. Schofield:** Funding acquisition; writing – review and editing; resources. **N. J. Menzies‐Gow:** Funding acquisition; supervision; writing – review and editing; resources.

## DATA INTEGRITY STATEMENT

A. Bouquet had full access to all the data in the study and takes responsibility for the integrity of the data and the accuracy of the data analysis.

## ETHICAL ANIMAL RESEARCH

This study was approved by the Social Science Research Ethical Review Board of the Royal Veterinary College, University of London (URN SR2022‐0166).

## INFORMED CONSENT

Informed consent was obtained for all participants in this study.

## Supporting information


**Survey S1.** Complete survey as uploaded to SurveyMonkey.

**Table S1.** Factors associated with HRQoL score in PPID and non‐PPID horses. Dependent variable: HRQoL score (SQRT). Df: degrees of freedom. Significant results in bold.

**Table S2.** Factors associated with HRQoL score in PPID horses. Dependent variable: HRQoL score (SQRT). Df: degrees of freedom. Significant results in bold.

## Data Availability

The data that support the findings of this study are available from the corresponding author upon reasonable request: Open sharing exemption granted by the editor.
