# Supplementary material for: Development of a health‐related quality‐of‐life assessment tool for equines with pituitary pars intermedia dysfunction
Source: Equine Vet J. 2025 May 2;58(1):190–202. doi: 10.1111/evj.14513 (PMC12699119; doi:10.1111/evj.14513)
Supplement: Supplementary file 1 — Survey S1. Complete survey as uploaded to SurveyMonkey. [file EVJ-58-190-s002.pdf]

Survey S1: Complete survey as uploaded to SurveyMonkey.

General information
Please tell us about your horse...

1. What is the name of your horse?
2. What breed is your horse?
3. How old is your horse?
4. How long have you owned your horse for?
5. How would you describe your horse's weight at the moment?
   a. Very overweight
   b. Slightly overweight
   c. Ideal weight
   d. Slightly underweight
   e. Very underweight
6. What sex is your horse?
   a. Mare
   b. Gelding
   c. Stallion
7. What type of livery do you have your horse on?
   a. DIY
   b. Part-livery
   c. Full-livery
8. Has your horse ever been diagnosed with PPID/Cushing's?
   a. Yes
   b. No
9. Has your horse been diagnosed with any other of the following chronic medical conditions (tick all that apply)
   a. Equine Metabolic Syndrome (EMS)
   b. Arthritis
   c. Recurrent Airway Obstruction (RAO)/equine asthma/heaves/COPD
   d. Skin condition e.g., sweet-itch
   e. Other (please specify):
10. Over his/her lifetime my horse has mostly been used for
    a. Competitions (e.g., racing, eventing, SJ dressage, endurance, showing or western?)
    b. Work horse (e.g., police)
    c. Hacking
    d. Field companion
    e. Human companion
    f. Other:
11. At this point in time, my horse is mostly used for
    a. Competitions (e.g., racing, eventing, SJ dressage, endurance, showing or western?)
    b. Work horse (e.g., police)
    c. Hacking
    d. Field companion
    e. Human companion
    f. Other:

12. I feel the overall quality of my horse's life is
    a. As good as it could possibly be
    b. Good
    c. Fairly good
    d. Neither good or bad
    e. Fairly poor
    f. Poor
    g. As poor as it could possibly be

Please answer the next section if your horse has been diagnosed with PPID/Cushing's disease, if not, this section can be skipped.

Your horse's PPID/Cushing's disease

13. When was your horse first diagnosed with PPID (approx., month and year)?
14. What veterinary-prescribed medication is your horse <u>currently</u> having for PPID?
    a. Prascend
    b. Pergoquin
    c. Pergolide paste
    d. Injectable cabergoline
    e. No veterinary-prescribed medication
    f. Other medication (please specify):
15. What veterinary-prescribed medication has your horse <u>had in the past</u> for PPID, since he/she was first diagnosed?
    a. Prascend
    b. Pergoquin
    c. Pergolide paste
    d. Injectable cabergoline
    e. No veterinary-prescribed medication
    f. Other medication (please specify):
16. Have you ever decided to take your horse off veterinary-prescribed medication for PPID?
    a. Yes
    b. No
17. Is your horse <u>currently</u> on any other non-veterinary prescribed treatments (e.g., supplements, homeopathy etc.) for PPID?
    a. Yes
    b. No
    If yes, which treatment(s):
18. Before my horse had PPID, his/her quality of life was
    a. A great deal better
    b. Quite a lot better
    c. A little better
    d. No different
    e. A little worse
    f. Quite a bit worse
    g. A great deal worse
    h. I don't know
19. Since my horse has been diagnosed with PPID, his/her quality of life is
    a. A great deal better
    b. Quite a lot better
    c. A little better
    d. No different
    e. A little worse
    f. Quite a bit worse
    g. A great deal worse
    h. I don't know

Now we are going to ask how your horse is feeling and looking <u>currently</u> (this month)

Demeanour/Behaviour

Please select which category best applies to how your horse is currently (this month)

20. My horse is dull, depressed and/or sad
    a. All the time
    b. Often
    c. Occasionally

    d. Never
21. My horse is spooky and unpredictable
    a. All the time
    b. Often
    c. Occasionally
    d. Never
22. My horse is moody
    a. All the time
    b. Often
    c. Occasionally
    d. Never
23. My horse has a lack of interest in life and/or is withdrawn
    a. All the time
    b. Often
    c. Occasionally
    d. Never
24. My horse enjoys engaging with other horses
    a. All the time
    b. Often
    c. Occasionally
    d. Never
25. My horse enjoys engaging with myself and other care takers
    a. All the time
    b. Often
    c. Occasionally
    d. Never
26. When my horse stands in the field, stable or tied up, his/her facial expression looks like a, b or c most of the time:
    a. 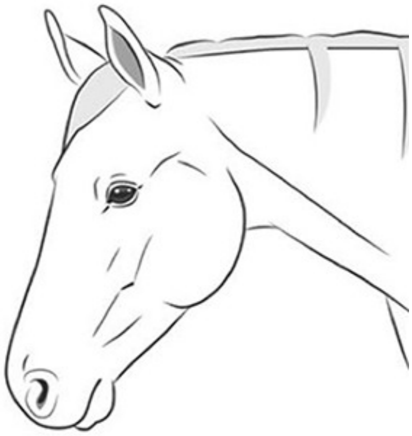

b. 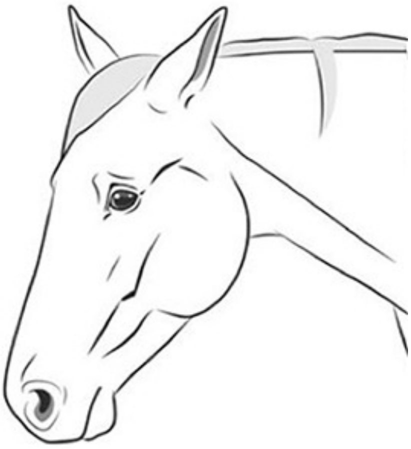

c. 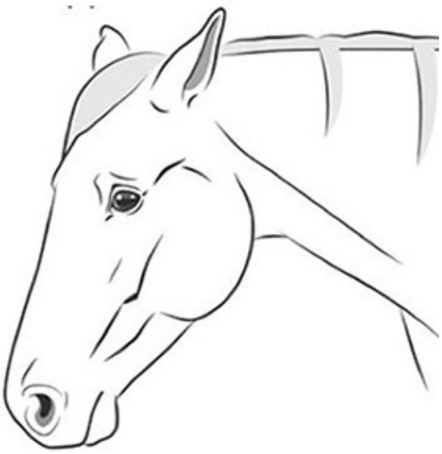

d. I haven't been able to observe this

Appearance

Please select which category best applies to how your horse is currently (this month)

27. My horse has a long and thick coat
    a. All the time
    b. Often
    c. Occasionally
    d. Never
28. My horse's coat is patchy
    a. All the time
    b. Often
    c. Occasionally
    d. Never
29. My horse's coat is bristly
    a. All the time
    b. Often
    c. Occasionally
    d. Never
30. My horse looks healthy
    a. All the time
    b. Often
    c. Occasionally

    d. Never
31. My horse looks older than he/she is
    a. All the time
    b. Often
    c. Occasionally
    d. Never

Condition
Please select which category best applies to how your horse is currently (this month)

32. My horse has lost their topline
    a. Strongly agree
    b. Slightly agree
    c. Neither agree or disagree
    d. Slightly disagree
    e. Strongly disagree
33. My horse has lost weight
    a. Strongly agree
    b. Slightly agree
    c. Neither agree or disagree
    d. Slightly disagree
    e. Strongly disagree
34. My horse has a cresty neck and other fat pocket deposits
    a. Strongly agree
    b. Slightly agree
    c. Neither agree or disagree
    d. Slightly disagree
    e. Strongly disagree
35. My horse has a big belly but is thin
    a. Strongly agree
    b. Slightly agree
    c. Neither agree or disagree
    d. Slightly disagree
    e. Strongly disagree

Health conditions
Please select which category best applies to how your horse is currently (this month)

36. My horse has laminitis
    a. All the time
    b. Often
    c. Occasionally
    d. Never
37. My horse is footy
    a. All the time
    b. Often
    c. Occasionally
    d. Never
    e. If all the time or often, any specific condition(s):
38. My horse has skin problems
    a. All the time
    b. Often
    c. Occasionally
    d. Never
    e. If all the time or often, any specific condition(s):
39. My horse has eye problems
    a. All the time

    b. Often
    c. Occasionally
    d. Never
    e. If all the time or often, any specific condition(s):

40. My horse has bad teeth
    a. yes
    b. no
41. My horse moves around freely and without pain
    a. All the time
    b. Often
    c. Occasionally
    d. Never

Diet and appetite
Please select which category best applies to how your horse is currently (this month)

42. My horse is fussy with and/or off his/her hard feed
    a. All the time
    b. Often
    c. Occasionally
    d. Never
    e. I haven't been able to observe this
43. My horse has no appetite (hay and other forage)
    a. All the time
    b. Often
    c. Occasionally
    d. Never
    e. I haven't been able to observe this
44. My horse drinks a lot
    a. All the time
    b. Often
    c. Occasionally
    d. Never
    e. I haven't been able to observe this

Ingestion of medication
Please select which category best applies to how your horse is currently (this month)

45. Is your horse on long-term medication prescribed by a vet?
    a. Yes
    b. No

    If yes, which medication(s):
46. Is your horse on any non-veterinary prescribed treatments or supplements?
    a. Yes
    b. No

    If yes, which treatment(s):

If your horse is not on daily veterinary prescribed medication or non-veterinary prescribed treatment, please skip to the next section.

47. I struggle to get my horse to eat the medication
    a. All the time
    b. Often
    c. Occasionally
    d. Never
48. My horse does not eat the medication in his/her bucket feed, I have to feed it in a treat or similar
    a. All the time

b. Often
c. Occasionally
d. Never

Management
Please select which category best applies to how your horse is currently (this month)

49. My horse needs clipping more often
    a. Strongly agree
    b. Slightly agree
    c. Neither agree or disagree
    d. Slightly disagree
    e. Strongly disagree
50. My horse lives a restricted life (e.g., feed restriction, restricted turnout etc.)
    a. Strongly agree
    b. Slightly agree
    c. Neither agree or disagree
    d. Slightly disagree
    e. Strongly disagree
51. My horse is still able to do all the activities that he/she used to enjoy and do
    a. Strongly agree
    b. Slightly agree
    c. Neither agree or disagree
    d. Slightly disagree
    e. Strongly disagree
52. I feel there is a strong bond between me and my horse
    a. Strongly agree
    b. Slightly agree
    c. Neither agree or disagree
    d. Slightly disagree
    e. Strongly disagree
53. Because of the care I give to my horse/my horse needs, my social/ working life is affected
    a. Strongly agree
    b. Slightly agree
    c. Neither agree or disagree
    d. Slightly disagree
    e. Strongly disagree
54. I struggle to manage my horse's health
    a. Strongly agree
    b. Slightly agree
    c. Neither agree or disagree
    d. Slightly disagree
    e. Strongly disagree
55. I worry about the future of my horse
    a. Strongly agree
    b. Slightly agree
    c. Neither agree or disagree
    d. Slightly disagree
    e. Strongly disagree
56. I worry about the health and wellbeing of my horse and have considered euthanasia
    a. Strongly agree
    b. Slightly agree
    c. Neither agree or disagree
    d. Slightly disagree
    e. Strongly disagree
57. I worry about the costs of the ongoing medical treatments that my horse needs to stay healthy
    a. Strongly agree

b. Slightly agree
c. Neither agree or disagree
d. Slightly disagree
e. Strongly disagree

Importance to you and your horse

Please rate the importance of the following issues to you and your horse with regards to their impact on quality of life, with 0 = not important at all and 10 = very important

1. Your horse's demeanour/behaviour

0, 1, 2, 3, 4, 5, 6, 7, 8, 9, 10

2. Your horse's appearance

0, 1, 2, 3, 4, 5, 6, 7, 8, 9, 10

3. Your horse's condition

0, 1, 2, 3, 4, 5, 6, 7, 8, 9, 10

4. Your horse's health condition

0, 1, 2, 3, 4, 5, 6, 7, 8, 9, 10

5. Your horse's diet and appetite

0, 1, 2, 3, 4, 5, 6, 7, 8, 9, 10

6. Your horse's ingestion of medication

0, 1, 2, 3, 4, 5, 6, 7, 8, 9, 10

7. Your horse's daily management

0, 1, 2, 3, 4, 5, 6, 7, 8, 9, 10
