# Supplementary material for: Development of a health‐related quality‐of‐life assessment tool for equines with pituitary pars intermedia dysfunction
Source: Equine Vet J. 2025 May 2;58(1):190–202. doi: 10.1111/evj.14513 (PMC12699119; doi:10.1111/evj.14513)
Supplement: Supplementary file 2 — Table S1. Factors associated with HRQoL score in PPID and non‐PPID horses. Dependent variable: HRQoL score (SQRT). Df: degrees of freedom. Significant results in bold. [file EVJ-58-190-s003.pdf]

Equine Veterinary Journal BEVA

Table S1: Factors associated with HRQoL score in PPID and non-PPID horses. Dependent variable: HRQoL score (SQRT). Df: degrees of freedom. Significant results in bold.

Variable		Type III Sum of Squares	df	Mean Square	F	Sig.
Intercept	Hypothesis	1.630	1	1.630	130.667	<0.001
	Error	0.426	34.132	0.012[a]		
**PPID diagnosis**	**Hypothesis**	**0.154**	**1**	**0.154**	**11.535**	**<0.001**
	**Error**	**7.749**	**579**	**0.013[b]**		
**Other chronic medical conditions**	**Hypothesis**	**0.220**	**1**	**0.220**	**16.429**	**<0.001**
	**Error**	**7.749**	**579**	**0.013[b]**		
Sex (binary)	Hypothesis	0.000	1	0.000	0.024	0.876
	Error	7.749	579	0.013[b]		
Breed	Hypothesis	0.073	6	0.012	0.907	0.490
	Error	7.749	579	0.013[b]		
Body condition	Hypothesis	0.077	4	0.019	1.446	0.217
	Error	7.749	579	0.013[b]		
Age (years)	Hypothesis	7.718E-6	1	7.718E-6	0.001	0.981
	Error	7.749	579	0.013[b]		
PPID diagnosis* Age (years)	Hypothesis	0.024	1	0.024	1.765	0.185
	Error	7.749	579	0.013[b]		
**Other chronic medical conditions * Age (years)**	**Hypothesis**	**0.079**	**1**	**0.079**	**5.926**	**0.015**
	**Error**	**7.749**	**579**	**0.013[b]**		
Sex (binary) * Age (years)	Hypothesis	0.001	1	0.001	0.110	0.740
	Error	7.749	579	0.013[b]		
Breed * Age (years)	Hypothesis	0.062	6	0.010	0.777	0.588
	Error	7.749	579	0.013[b]		
Body condition * Age (years)	Hypothesis	0.032	4	0.008	0.607	0.658
	Error	7.749	579	0.013[b]		

a. 0.146 MS(Sex_binary) + 0.168 MS(Breed) + 0.201 MS(Weight) + 0.485 MS(Error)
b. MS(Error)
