# Supplementary material for: Development of a health‐related quality‐of‐life assessment tool for equines with pituitary pars intermedia dysfunction
Source: Equine Vet J. 2025 May 2;58(1):190–202. doi: 10.1111/evj.14513 (PMC12699119; doi:10.1111/evj.14513)
Supplement: Supplementary file 3 — Table S2. Factors associated with HRQoL score in PPID horses. Dependent variable: HRQoL score (SQRT). Df: degrees of freedom. Significant results in bold. [file EVJ-58-190-s001.pdf]

Table S2: Factors associated with HRQoL score in PPID horses. Dependent variable: HRQoL score (SQRT). Df: degrees of freedom. Significant results in bold.

Variable		Type III Sum of Squares	df	Mean Square	F	Sig.
Intercept	Hypothesis	0.768	1	0.768	50.748	<0.001
	Error	0.248	16.382	0.015[b]		
Current PPID treatment (binary)	Hypothesis	4.706E-5	1	4.706E-5	0.003	0.956
	Error	4.497	288	0.016[c]		
**Other chronic medical conditions**	**Hypothesis**	**0.081**	**1**	**0.081**	**5.182**	**0.024**
	**Error**	**4.497**	**288**	**0.016[c]**		
Sex (binary)	Hypothesis	0.013	1	0.013	0.865	0.353
	Error	4.497	288	0.016[c]		
Breed	Hypothesis	0.074	6	0.012	0.791	0.578
	Error	4.497	288	0.016[c]		
Body condition	Hypothesis	0.074	4	0.018	1.179	0.320
	Error	4.497	288	0.016[c]		
Age (years)	Hypothesis	0.001	1	0.001	0.074	0.786
	Error	4.497	288	0.016[c]		
Current PPID treatment (binary)* Age (years)	Hypothesis	0.003	1	0.003	0.215	0.643
	Error	4.497	288	0.016[c]		
Other chronic medical conditions * Age (years)	Hypothesis	0.027	1	0.027	1.726	0.190
	Error	4.497	288	0.016[c]		
Sex (binary) * Age (years)	Hypothesis	0.003	1	0.003	0.177	0.675
	Error	4.497	288	0.016[c]		
Breed * Age (years)	Hypothesis	0.064	6	0.011	0.680	0.666
	Error	4.497	288	0.016[c]		
Weight * Age (years)	Hypothesis	0.053	4	0.013	0.854	0.492
	Error	4.497	288	0.016[c]		
Other chronic medical conditions * Years diagnosed	Hypothesis	0.001	1	0.001	0.036	0.850
	Error	4.497	288	0.016[c]		
Current PPID treatment (binary) * Years diagnosed	Hypothesis	0.009	1	0.009	0.590	0.443
	Error	4.497	288	0.016[c]		
Sex (binary) * Years diagnosed	Hypothesis	0.012	1	0.012	0.749	0.387
	Error	4.497	288	0.016[c]		
Breed * Years diagnosed	Hypothesis	0.023	6	0.004	0.243	0.962
	Error	4.497	288	0.016[c]		
Body condition * Years diagnosed	Hypothesis	0.013	4	0.003	0.205	0.935
	Error	4.497	288	0.016[c]		

a. PPID diagnosis = yes
b. 0.198 MS(Sex_binary) + 0.233 MS(Breed) + 0.251 MS(Weight) + 0.317 MS(Error)
c. MS(Error)
